# Phenotypic variability and community interactions of germinating *Streptomyces* spores

**DOI:** 10.1038/s41598-017-00792-7

**Published:** 2017-04-06

**Authors:** Ye Xu, Kalin Vetsigian

**Affiliations:** Department of Bacteriology and Wisconsin Institute for Discovery, University of Wisconsin-Madison, Wisconsin, 53715 USA

## Abstract

A case can be made for stochastic germination and interactions among germinating spores as beneficial germination strategies in uncertain environments. However, there is little data on how widespread, species-specific or diverse such phenomena are. Focusing on Streptomycetes, a platform was developed for quantification of germination and early growth within communities of spores. We found that the germination process is stochastic at three levels: spores vary in their germination times, mycelium networks grow at different rates, and a fraction of germlings stall their growth shortly after germination. Furthermore, by monitoring how these stochastic properties are affected by spore density and chemicals released from spores, germination interactions were quantified for four species. Stochastically germinating spores were frequently promoted or inhibited by compounds released by spores from the same or different species, and all species had distinct interaction profiles. The spatial distribution patterns were important with clusters of spores behaving differently than individual spores. Aged spores exhibited higher dormancy but could efficiently geminate in the presence of chemicals released during germination. All interactions were specific to germination and only weakly affected growth rates. This work suggests that stochastic germination is commonly affected by the community context and species have adapted diverse germination strategies.

## Introduction

In most natural habitats bacteria live in crowded and diverse communities. Correspondingly, they have evolved social traits^1–3^. For example, microbes can affect their neighbors by secreting antibiotics^1–5^, or quorum sensing molecules^1–6^. But, judging by the vast genomic potential for small molecule production^1–10^, we are far from having explored the full range of social capabilities of microbes, let alone their evolutionary and ecological implications.

Bacterial germination is an area in which social phenomena have been poorly explored. A dormancy phase is ubiquitous among bacteria, fungi and plants, and in many environments a large fraction of the microbes exist as spores^1–5, 11^. When to germinate is a fateful decision because if the environmental conditions turn out to be unsuitable for growth a germinated spore might die before it has a chance to undergo a life cycle leading to more spores. One strategy for making this decision is environmental sensing in which spores detect the onset of a favorable environment. This strategy is complicated by the fact that bacterial growth requirements are typically complex and detection of no single molecule can guarantee success. At the same time, monitoring multiple environmental variables and performing complex calculations might be prohibitively expensive, or even impossible, for dormant spores^6, 12^. Even if perfect sensing were possible, deterministically triggered germination can lead to disastrous outcomes if the favorable environment does not persist. This is because it effectively induces all spores in an environment to behave in the same way.

Theoretical studies suggest that stochastic switching could be favored to environmental sensing^13, 14^ in uncertain environments, and in fact stochastic germination has been identified^15^ and recently investigated^11, 16^ in *Bacillus subtilis*. But stochastic germination can be problematic as well, at least in its simplest form, because it sets a time scale for survival in an unsuitable environment, while the duration of unsuitable environments might be prone to large fluctuations. Even if the duration of unsuitable environments has a typical time scale to which the stochastic process can be tuned, a slow stochastic switching from dormancy to growth can be disadvantageous in a competitive environment in which the first to germinate and grow has a large competitive advantage.

But what else can spores do? An intriguing possibility is that stochastic germination decisions are informed by neighboring bacteria. Such collective germination strategies might be superior to individual based strategies in uncertain environments. Fascinatingly, a case can be made for either inhibition or promotion of germination as an adaptive strategy.

On one hand, inhibition of neighbors by chemicals secreted by germinating spores might be evolutionary favored because it would provide a local competitive advantage during the critical period of early colonization^17^. Moreover, self-inhibition within a species can act as a coordinated bet-hedging strategy. In case conditions are bad, all sister spores in the vicinity will be saved from germinating; if conditions are good, there will be hardly any harm in inhibiting sister spores in close proximity since the colony from the first spore is already exponentially growing. Such a strategy can allow frequent probing of the environment by spontaneously germinating spores, thus increasing the probability that spores from a given species are the first to initiate growth when conditions are good, without risking germination of all spores in a population within a short time period if conditions are bad. Two compounds have been isolated from *Streptomyces* spores that can inhibit germination within species^11, 18–22^. Germicidin is released upon germination of at least two species and potentially fits the spore-communication framework. The second compound, hypnosin, seems to be released from the inside or surface of spores as soon as they are wetted and before germination, and thus might serve as a mechanism to prevent spores from germinating after sporulation until they are washed away from their parental context.

On the other hand, one might expect germination promoting interactions. Spores might have evolved to detect byproducts of growing bacteria as an indication that conditions are good and germinate in response. Thus, one can imagine a scenario in which spontaneous germination rates are kept very low because favorable environments are unlikely, but positive interactions lead to germination blooms triggered by growing bacteria. By the same token dedicated self-promotional signals can evolve within a species as a form of cooperative behavior – a small fraction of spores germinate to scout the environment and if conditions are good wake up other spores from the same species^23^. Self-promotion might be particularly favored if an Allee effect, in which microbial growth rates increase with population size, is operational. This might be the case for example if a public good in the form of an extracellular enzyme or siderophore is needed for growth. Several lines of evidence support the importance of germination promotion interactions. The germination of spores of *Bacillus subtilis* is triggered by peptidoglycan fragments (muropeptides), which are a byproduct of bacterial growth^24, 25^. A secreted protein known as resuscitation-promoting factor (Rpf) has been found to resuscitate spores of *Micrococcus luteus* ^26^ and *M. tuberculosis* ^27^. Recently, Rpf proteins were also shown to be important for germination in *Streptomyces coelicolor* ^28^. Higher-order interactions are also possible: staurosporine secreted by *Streptomyces staurosporeus* can block the muropeptide-promoted spore germination^24^.

Despite these fascinating examples, it is currently unknown how widespread or diverse microbial germination interactions are. It is unknown whether they are predominantly positive or negative, whether they are broad-spectrum or specific, and whether interactions between spores of the same strain are typical or not. There has also been no systematic investigation of how widespread stochastic germination is, and how the stochastic properties of germination interplay with intercellular communication.

A factor limiting the accumulation of knowledge in this area has been the stochastic nature of the germination processes. Most previous germination experiments have been performed by periodic subsampling followed by manual microscopic observation and scoring, or by measurements of population averages through proxies such as the decrease of the optical density of activated spores in liquid. Bacterial germination has not been previously quantified in a context where spatial structure is preserved or at the single - cell level.

**Figure 1:**
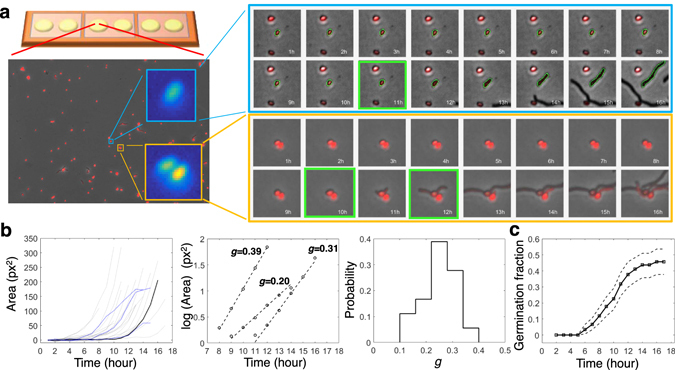
Platform for high-throughput quantification of phenotypic variability of germination and early growth. (**a**) A device containing multiple agar wells was used to monitor germination of fluorescent spores over time in parallel communities. Up to 40 fields of view per well were imaged for up to 24 hours. Individual spores were identified and, if clustered, segmented based on their fluorescent intensity. Shown are time-lapse images for an individual spore (blue, top) and a pair of touching spores (orange, bottom). The outlines of spores and emerging mycelium tubes were identified through image processing (green contours). The germination time was determined as the onset of spore elongation (green frame). (**b**) Analysis of initial growth of individual spores. (Left) Plot of area growth as a function of time reveals a lag phase followed by an exponential growth phase (the individual spore from (**a**) is shown in dark black). In some cases, the growth of mycelium stalled (blue). (Middle) Initial growth rate, *g*, was deduced by fitting a line to log(area) versus time. (Right) The distribution of growth rates of all growing spores from the field of view shown in (**a**). (**c**) The cumulative fractions of germinated spores over time for all 151 spores from the field of view shown in (**a**). Dashed curves indicate the 95% confidence interval for the germination curve.

Focusing on bacteria from the genus *Streptomyces*, which are prolific small - molecule producers, we have developed a novel high-throughput platform for automated quantification of spore germination on agar surfaces at the single-cell level (Fig. 1). Our platform provides a full picture of germination events and subsequent early growth for each of many thousands of individuals for multiple communities in parallel. Each individual spore is characterized by its coordinates, germination time, initial rate of mycelium growth and whether or not growth stalls after germination. Assays with fluorescently labeled spores allow us to perform highly automated image analysis in single- and two-species communities of spores.

This methodology, enabled us to quantify the stochastic properties of spore germination and early growth and investigate how these stochastic properties are influenced by the community context. In particular, we asked how germination is affected by spore density, spore clustering, chemicals released after germination from same and different species, and presence of spores from other species.

## Results

### A high-throughput platform for quantifying phenotypic variability of germination and early growth of *Streptomyces*

A silicone device containing 6 individual wells filled with thin agar layers was used to monitor germination of fluorescent spores over time in parallel communities (Fig. 1a). Time-lapse imaging of germination and mycelium growth was performed over 24 hours for 10–40 fields of view within each well. Custom scripts were used to register the outlines of spores and the emerging mycelium networks over time (Fig. 1a, blue-framed movie strip). This data was used to determine germination and growth rates over time (Fig. 1b). Germination time was defined as the onset of increase of the length of the major axis of spores. The growth of mycelium was quantified based on the area (number of pixels) occupied by mycelium contours over time. Given that the mycelium thickness is roughly constant, this area is proportional to the total length of the mycelium filaments within a network. The mycelium area typically increased exponentially over time, which allowed us to assign growth rates to germinated spores (Fig. 1b, Fig. S1). A fraction of mycelium networks stopped growing soon after germination (Fig. 1b left, blue lines, Fig. S1a), an outcome we termed *stalling*. Fluorescent labeling of spores allowed us to reliably delineate individual spores within clusters by using a watershed algorithm (Fig. 1a, orange-framed movie strip). At the single-cell level, we characterized spores by their coordinates, germination times, growth rates, whether they stalled or not, and whether they were part of a cluster or not. At the population level, we calculated: germination curves expressing the cumulative fraction of germinated spores as a function of time (Fig. 1c), the distribution of growth rates (Fig. 1b right), and the fraction of stalling mycelium networks among germinated spores. In this way, the platform allows us to characterize the phenotypic variability within germinating communities of spores.

### Germination is a Stochastic Process which Exhibits Qualitative Differences between *Streptomyces* species

**Figure 2:**
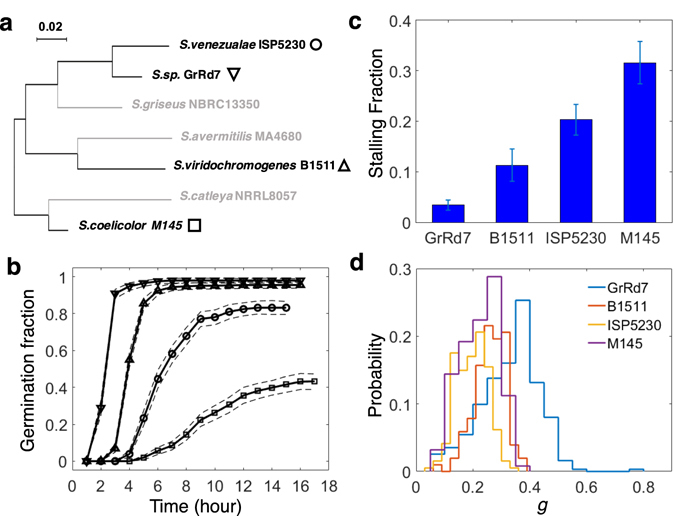
Germination and growth of spores of four *Streptomyces* species. (**a**) Phylogenetic relationship of the 4 investigated *Streptomyces* species. (**b**) The germination curves of the 4 species. The curves differ in the onset of germination, germination rate, and final fraction of germinated spores. Dashed curves show 95% confidence intervals. (**c**) Shown is the fraction of germlings that stopped growing soon after germination for the dataset in (**b**). Error bars indicate 95% confidence intervals. (**d**) Shown are the distributions of mycelium growth rates for the four strains. All the data was taken with the same media, spore concentration, and spore age. See additional figure data in Methods.

We fluorescently labeled the spores of 4 Streptomyces strains (Table S1), which were not closely related within the genus (Fig. 2a). We started by comparing their stochastic germination properties within monocultures of spores of the same density and age on diluted nutrient broth media. The germination curves of the species differed in terms of their germination rates and qualitative properties (Fig. 2b). Two of the strains (*S. sp* GrRd7 and *S. viridochromogenes* B1511) exhibited robust germination with close to 100% of the spores germinating (Figs 2b and S2). The curves were characterized by a lag phase followed by a sudden jump in germination rates that then monotonically decreased in a manner which was quantitatively consistent with a simple constant germination probability per spore per unit time model (exponential decay of non-germinated fraction) (Fig. S2). The robust germination behavior of these two strains persisted at lower nutrient concentrations, and even on purified agar without addition of any resources (Fig. S3a,b). In contrast, the other two strains (*S. venezualae* ISP5230 and *S. coelicolor* M145) exhibited more complex germination behavior characterized by a fraction of spores that does not germinate even after an extended wait (Fig. 2b) and by germination rate per spore that does not monotonically change over time (Fig. S4). Both of these qualitative properties persist as we decrease the spore density (Fig. S4), which suggests that they are not emerging dynamically from inter-cell interactions (e.g. accumulation of germination inhibitors in the media) but likely result from pre-existing differences between spores or multi-step stochastic processes within individual spores. We also found that the non-germinating fraction of spores is not due to the lack of viability. In fact, the fraction of germinated spores can be increased by increasing the concentration of resources in the media (Fig. S3c,d). This suggests that spores have a wide pre-existing variability in the minimal nutrient concentrations they require for germination, which might for example result from variability in the number of receptor proteins or transcription factors^16^.

### Bistability of Fates After Germination

The variability in behaviors was not restricted to the germination phase. All investigated strains exhibited bistability of growth behavior after germination (Fig. S5a). While some germlings gave rise to exponentially growing mycelium colonies, the growth of other germlings slowed down and stalled. Such phenomenon suggests that even in environments permissive for growth there can be a risk associated with the germination and growth initiation process. The stalling of germlings also implies that macroscopic colony counts cannot be reliably used to accurately assess germination rates within the genus *Streptomyces*.

### Nascent mycelium networks grow exponentially but at individual-specific growth rates

For most nascent mycelium networks, the increase of the total length of mycelium filaments (image area covered by mycelium) after germination was exponential with a very high accuracy (Fig. 1b middle). The growth was exponential from the onset of germination and remained so as the mycelium started to branch. This is consistent with previous studies showing that right after germination the extension of the unbranched mycelium is accelerating^29, 30^ before settling into a regime in which the extension of individual hyphae becomes linear but the overall growth of the network is exponential because of branching^31, 32^. Strikingly, while the individual networks grew exponentially in a deterministic fashion, different individuals varied greatly in terms of their growth rates. The growth rates could vary up to three-fold (Fig. 2d). The growth rate was largely uncorrelated with germination time (Fig. S5b), which indicates that growth rate differences cannot be explained by exhaustion of resources slowing down the growth of late germinating spores. The lack of correlation also indicates that the stochastic processes determining germination and growth are largely independent.

### Interaction among Germinating Spores in Single-species Communities

**Figure 3:**
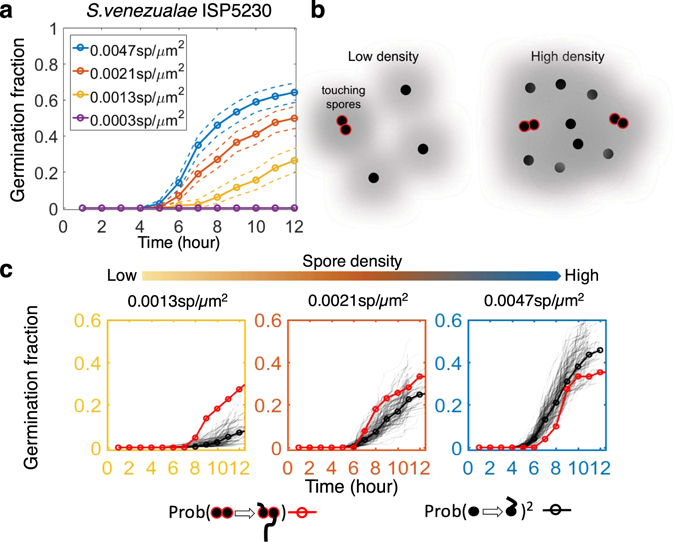
Self-promotion of germination among neighboring *S. venezualae* ISP5230 spores. (**a**) Germination curves at different spore densities. Spores germinate earlier and more spores germinate at higher density, suggesting that germinating spores promote germination of sibling neighbors. 95% confidence intervals are indicated by dashed lines. All differences between curves are significant (*p* < 10^−5^). (**b**) Spores are hydrophobic and naturally clustered as touching spores (red outlined spores) at any density. If an interaction exists between spores and the average distance between spores is larger than the interaction range (grey halo around spores), the germination probabilities of touching spores can be different from those of single spores. (**c**) The observed probability that both touching spores in a doublet germinate by some time (red curve) is compared with the expectation from a null model assuming that touching spores germinate independently according to the probability distribution for single spores (black). Thin black curves show a hundred random realizations for doublets germinating as singles. The thick black curve shows the averaged expectation. The three panels correspond by frame color to the different spore densities in (**a**). The leftmost panel shows a higher germination probability of touching pairs than that expected based on isolated spores (*p* = 0.0022). See additional figure data in Methods.

One way to look for germination interactions within species is to compare germination curves at different spore densities (Figs 3a and S6). If the overall germination probabilities are higher when the spores are closer together, this would indicate a self-promotion of germination. On the other hand, if the germination probabilities are lower at higher spore density, this would indicate self-inhibition. We compared the relation between germination curves and spore densities for each of the four *Streptomyces* strains. The germination of *S. venezualae* ISP5230 showed a strong positive correlation with its spore density (Fig. 3a) and fit into the first category. *S. coelicolor* M145 had a slight reduction of the germinated fraction at higher density (Fig. S6c). However, it manifested itself only after accumulation of mycelium and might be at least partially due to decreased amount of resources. Lastly, GrRd7 germination was not significantly and reproducibly affected by the spore density (Fig. S6a), and B1511 had its germination slightly delayed at lower density (Fig. S6b).

### Spatial Scale of Self-promotion

By investigating the spatial correlations among germinating spores we can evaluate the effective range of interactions. If the molecules affecting germination diffuse very fast then every spore would feel pretty much the same influence, which would lead to no discernable spatial structure. On the other hand, if diffusion is slow then spores should germinate more near other germinating spores for the self-promoting strain *S. venezualae* ISP5230.

A feature we exploited was that clusters of two or more touching spores were present for a wide range of spore densities (Fig. S11). This allowed us to compare germination within pairs of touching spores (doublets) to that of individual spores (Fig. 3b). In particular, we asked if the germination curve for isolated spores, ${P}_{single}(t)$, is predictive of germination of spores in doublets. If ${P}_{single}(t)$ is the germination curve for singles, then ${P}_{single}^{2}(t)$ is the probability that two random spores both germinate by time $t$. We can compare this null expectation with the probability that two touching spores both germinate. We applied this analysis to the community of *S. venezualae* ISP5230 spores (Fig. 3c), and found that doublets germinate better than singles only when the density is low (left panel, *p* = 0.0022, see Methods). At medium density the promotional effect disappeared (mid panel, *p* = 0.16), and if anything was replaced by a slight inhibition at high density (right panel, *p* = 0.94), possibly due to local competition for resources. From this, we conclude that the effective range of the self-promotion of *S. venezualae* ISP5230 is on the order of 10 µm under these experimental conditions. Clusters of three or more spores were excluded from this analysis.

### Effect of Chemicals Released at the Onset of Germ Tube Emergence

**Figure 4:**
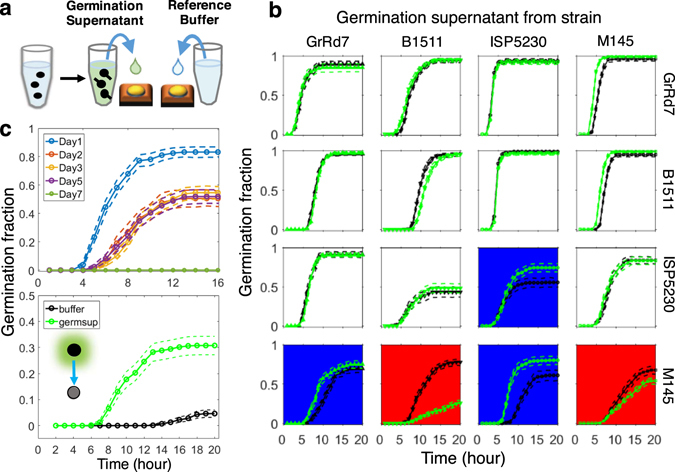
Supernatant experiments reveal the existence of positive and negative, intra- and inter- species germination interactions. (**a**) Spores were grown in nutrient broth until a fraction of spores formed short germ tubes. This germination supernatant was applied to agar-based spore populations, which were compared to ones threated with a reference buffer. (**b**) Fresh spores from one species were treated by a germination supernatant from another species (green lines) or a reference buffer (black lines). The reference buffer shown is H_2_O for cases with germination inhibition (red background) or the nutrient broth for cases with germination promotion (blue background) or no interaction (white background). All highlighted interactions are significant, *p* < 10^−4^. (**c**) Germinability of ISP5230 spores drops dramatically with storage time (top). But supernatant from fresh spores restores the germinability of old spores (bottom). (**b**,**c**) Dashed lines indicate 95% confidence intervals for each germination curve. See additional figure data in Methods.

We then examined whether the self-promotional activity is present when spores germinate in liquid media (Fig. 4a). We applied the supernatant from a liquid culture at the onset (~4 h for *S. venezualae* spores) of germ tube formation (*germsup*) to spore communities inoculated on agar. We compared the germination curves between spore communities treated by the germination supernatant and reference buffers consisting of the liquid media in which spores germinated or H_2_O. The amount of resources added with the liquid media equaled 1/80 of those in the agar wells for most experiments. Consistent with the experiments at different densities, *S. venezualae* ISP5230 spores growing on agar supplemented by its own germsup germinated significantly faster than either reference (Fig. 4b, 3^rd^ row and 3^rd^ column). Germination supernatant from spores incubated for only 1.5 h, way before the emergence of the first germ tubes was observed in the population, also exhibited some, though much weaker, promotional activity (Fig. S7a) — indicating that secretion of the promotion germination factor precedes visible germ tube formation but is also linked to germination.

We also tested the self-interactions of the other three *Streptomyces* species using their respective germination supernatants and got results consistent with those obtained by comparing germination curves at different spore densities (Fig. 4b, diagonal panels). The mild self-inhibition on germination of *S. coelicolor* M145 was confirmed by its own germsup, which indicated that reduction we saw at high density is not due to resource competition – germsup supplemented media had lower germinability even than a water supplemented control despite the extra nutrients. Moreover, increasing the concentration of nutrients by five-fold in the liquid in which the supernatant was made led to a stronger inhibition despite the extra nutrients (Fig. S8). The germsups of *S.sp* GrRd7 and *S. viridochromogenes* B1511 had no effect on their own germinations. Lack of self-inhibition was surprising for *S. viridochromogenes* B1511 given previous reports that its spores contain germicidin which is a potent germination inhibitor^19, 20^. The media used for sporulation and germination in our experiments were different, giving rise to the intriguing possibility that spores made on different media or germinating in different circumstances can manifest different interactions.

### Self-promotion Chemicals can Restore Germinability of Old Dormant Spores

The germinability was influenced by the amount of time spores spent in the Tris storage buffer without nutrients (Figs 4c and S9). The strain *S. venezualae* ISP5230 was the most significantly affected (Fig. 4c, top), and its germinability dropped dramatically in only a few days. These ‘old’ spores were not dead but had entered into a state of deeper dormancy that required enhanced nutrient levels for germination (Fig. S10a). Strikingly, germsup from fresh spores completely restored the germinability of old spores to levels matching those of younger spores (Fig. 4c, bottom). The dormancy of old spores could not be accounted for by leakage of the promotional factor during storage since no activity was recovered from the prolonged storage buffer (Fig. S7b). In addition, as the self-promotional activity disappeared after 10 min at 100 °C (Fig. S10b), it is unlikely that this self-promotional activity is due to cell wall debris (muropeptides), which was previously shown to trigger exit from dormancy in some bacteria^25^. This analysis does not rule out a potential role of secreted Rpf proteins^28^.

### Interspecies Germination Interactions

Extending the germsup experiments, we quantified the germination interactions between pairs of different species for the panel of all four investigated Streptomycetes (Fig. 4b). Interestingly, germsup from *S. viridochromogenes* B1511, which did not affect germination of its own spores, showed a potent inhibition of germination of *S. coelicolor* M145 and a weak promotion of *S. venezualae* ISP5230. Thus, chemicals from one strain can have qualitatively different effects on other strains. Similarly, germsup of *S.sp*. GrRd7 had no effect on itself but showed mild promotion of *S. coelicolor* M145 at early time points. Germsup of *S. venezualae* ISP5230 not only self-promoted but also strongly promoted *S. coelicolor* M145. The two robustly germinating species *S.sp*. GrRd7 and *S. viridochromogenes* B1511 were not significantly affected by any germsup.

### The Effect on Mycelium Growth of Compounds Promoting or Inhibiting Germination

**Figure 5:**
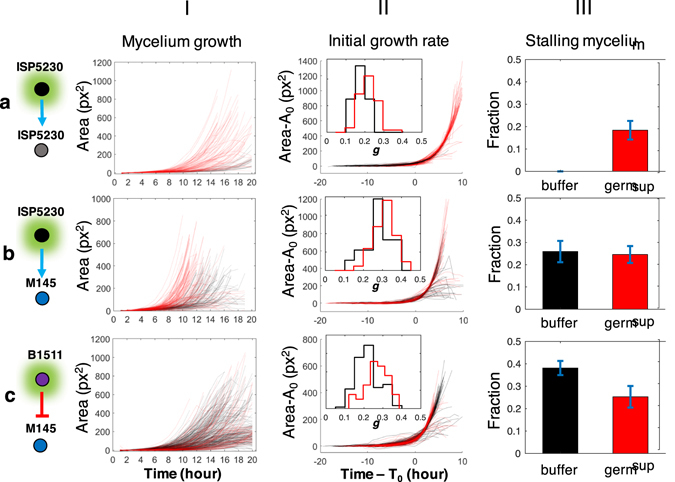
The effect of chemicals in germination supernatants on mycelium growth. The growth curves (I), the distribution of growth rates (II), and the stalling fractions (III) were compared between spores treated with germsup (red) or reference buffer (black) for three germination interactions shown in Fig. 4. The shown reference buffer is nutrient broth for the positive interactions and water for the negative ones. The area curves in (II) were aligned by shifting them along the time and area axes in order to compare growth after germination. The distribution of growth rates (*g*) is shown in the inset. (**a**) The germsup from fresh spores of ISP5230 that strongly promoted germination of aged ISP5230 spores (Fig. 4c) mildly increased growth rates (II) (p = 0.009). The fraction of stalling mycelium treated by reference buffer could not be determined due to the much-delayed germination (III). (**b**) The germsup of ISP5230 promoting the germination of M145 (Fig. 4b) did not affect the growth rate (II) or stalling fraction (III) of the M145 mycelium. (**c**) The germsup of B1511, which inhibits the germination of M145 (Fig. 4b), slightly but significantly increased growth rate (II) (p = 3 × 10^−6^) and decreased the stalling mycelium fraction of M145 (III) (p = 0.037).

We examined whether the interactions we observed are specific to germination or whether they also promoted or inhibited mycelium growth after germination (Fig. 5). The germination supernatant of *S. venezualae* that restored the germinability of its aged spores (Fig. 5a) slightly but significantly increased the initial growth rates of mycelium emerging from the old spores (Fig. 5a(II)). However, the *S. venezualae* germsup, which also promoted the germination of *S. coelicolor*, had no effect on the distribution of growth rates or the stalling fraction of mycelium (Fig. 5b(II and III)). The germsup of *S. viridochromogenes*, which inhibits the germination of *S. coelicolor*, did not inhibit growth after germination. If anything, this germsup promoted growth after germination, leading to significantly less stalling mycelium and higher growth rates (Fig. 5c(II and III)). This data demonstrates that chemicals that promote or inhibit germination do not necessarily affect growth after germination and can even have opposite effects on germination and growth.

### Interactions in Two-species Communities

We proceeded to determine if the interactions observed in germination supernatant experiments manifest themselves in the context of two-species communities. The interactions can be different in the two cases because germination in liquid is different than germination on agar or because of phenomena specific to co-culturing. For example, the secretion and accumulation of compounds from one of the species can be too slow to affect the other, or there might be bidirectional communication between the species inducing different interactions. Still given that secretion of compounds starts early on, one might reasonably expect that supernatant interactions are often predictive of what happens in two-species communities.

**Figure 6:**
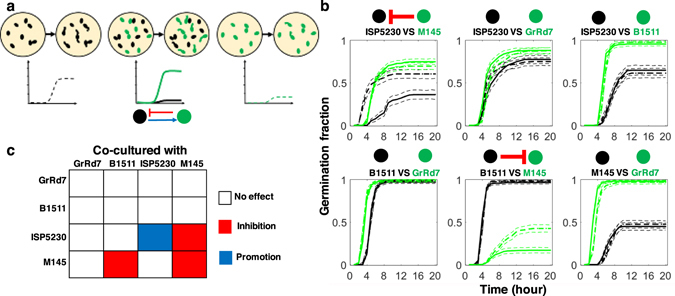
Germination interactions in two-species communities. (**a**) Schematic of the experiment. Red fluorescent spores (shown in black) and green fluorescent spores (shown in green) from different strains are grown by themselves (dashed germination curves) or together (solid germination curves). (**b**) The results from the six 1:1 co-cultures of two different strains are shown along with the inferred interactions. The 95% confidence intervals are indicated as thin dashed lines. All sample sizes are above 200 spores. ISP5230 is inhibited by M145 (*p* < 10^−26^). M145 is inhibited by B1511 (*p* < 10^−15^). (**c**) The matrix of germination interactions deduced through co-culture experiments.

We co-cultured differentially labeled spores of different species in 1:1 ratio and quantified germination curves for each species in the mixed spore community (Fig. 6a). We compared these to the germination curves of the species growing by themselves with each species kept at the concentration it had in the two-species community. This scheme controls for the density dependence of germination for *S. venezualae* ISP5230 and *S. coelicolor* M145 and was further validated by the fact that it left most germination curves the same between the single- and two-species communities.

Performing all pairwise combinations we discovered only two pronounced inter-species interactions (Fig. 6b): One was the inhibition on germination of *S. coelicolor* M145 by *S. viridochromogens* B1511, which is consistent with our finding in the germsup experiment. The other was a strong repression on *S. venezualae* ISP5230 by *S. coelicolor* M145, which we did not see in the germsup experiments. This difference with the germsup data cannot be attributed to *S. coelicolor* M145 outgrowing *S. venezualae* ISP5230 because the latter germinates earlier than the former in isolation and because germination of *S. venezualae* ISP5230 was strongly suppressed well before a significant accumulation of mycelium. We then tested germsup from co-incubation of both strains in liquid and found that it had promotional rather than inhibitory activity on *S. venezualae* ISP5230. Periodic microscopic observation of spores of *S. coelicolor* M145 incubated in liquid revealed that their germination on agar was much faster and more robust compared to that in liquid. Thus, the presence of a solid surface or micronutrients from agar alter the behavior of spores of *S. coelicolor* M145, which likely leads to an increased inhibitory activity. This difference between germsup and co-culture experiments highlights the context dependence of germination interactions and their sensitivity to apparently small differences in environmental conditions.

In contrast to the inhibitory interactions and the self-promotion of *S. venezualae* ISP5230, the other inter-species promotional interactions noted earlier did not manifest themselves in the context of two-strain communities. The lack of promotion of *S. coelicolor* M145 by *S. venezualae* ISP5230 can be understood in terms of a faster acting inhibition of the latter by the former that delays the release of the promotional activity. On the other hand, the loss of the other two promotional interactions is likely due to the fact that the bacteria secreting the germination promotion compounds germinated earlier and grew substantially more robustly than their partners, thus offsetting any positive effect through increased resource competition. This data demonstrates that the chemical ability of compounds from one spore species to affect others needs to be coupled with suitable relative germination and growth rates in order to be ecologically important.

Overall, interactions from single-species experiments at different densities and co-culture experiments significantly overlap with interactions deduced from supernatant experiments and confirm the finding that interactions are common and can be both positive and negative (Figs 4b and 6c). Furthermore, they demonstrate the ecological relevance of germination interactions for populations of spores growing on surfaces.

## Discussion

We developed an experimental platform for quantifying germination and early growth in communities of spores and harnessed it to investigate the germination behavior and interactions of four *Streptomyces* species. Two of the four species exhibited robust germination, while two species displayed complex stochastic germination behavior in which only a subsets of the spores germinated. Interestingly, the two robustly germinating species were never affected by chemicals released by spores (even inhibitions), whereas the stochastically germinating species were frequently promoted or inhibited. This suggests a link between stochastic germination behavior and sensitivity to early released compounds and is consistent with the hypothesis that spores combine stochastic germination behavior with positive or negative interactions as an adaptive germination strategy in uncertain environments. It would be important to examine this potential link across more strains or across many media for the same strain.

Basic questions we investigated were whether germination interactions are diverse and whether some particular type of germination interaction is much more common than the others. The dataset we generated, though limited in terms of number of species, already revealed the existence of all types of interactions. We found that both intra- and inter- specific interactions are common and that both can be either positive or negative. Furthermore, compounds released by the spores of a species can have qualitatively opposite effects on other species, as exemplified by the extract from spores of *S. viridochromogenes* B1511 which inhibits *S. coelicolor* M145 and promotes strain *S. venezualae* ISP5230. Thus, spores of different strains can interpret the same molecular cocktail differently. This, together with the fact that all strains could be distinguished based on the effects of compounds released by their spores, highlights the evolutionary plasticity of germination interactions. Equally fascinating is the finding that the same strain can be either promoted or inhibited depending on the microbial context, as demonstrated by *S. coelicolor* M145 in the supernatant experiments and by *S. venezualae* ISP5230 in the co-culture ones. Overall, the network of germination interactions exhibited maximal diversity and complexity, which we hope will stimulate further research in this area.

Beyond the interaction network, our work pointed at several additional levels of complexity. First, *Streptomyces* spores change with age and can exhibit different levels of dormancy. These different states can have different stochastic behavior and different responses to germination signals. Particularly, we discovered that the stochastic behavior of *S. venezualae* ISP5230 spores sharply changes with age and the effect of self-promotion is more pronounced for older spores. Promoted old spores germinated similarly to fresh spores. Second, since compounds need to diffuse between spores, the spatial distribution patterns of spores are important. We found that clusters of spores germinate differently than isolated spores. Thus, the facts that *Streptomyces* spores arise in chains that disperse imperfectly and that spores tend to clump due to hydrophobicity might be ecologically significant. Finally, the unidirectional interactions we determined through germination supernatant experiments were just one aspect of a complex dynamic process and did not always predict the qualitative outcomes in two-species communities. In particular, under the experimental conditions examined we could not detect a significant promotional effect between different strains. It would certainly be of great interest to further explore whether different spore ages, species ratios, spore densities or environmental conditions would allow for inter-species promotion to be manifested. A fuller utilization of the spatial and temporal correlations within the germination and early growth data that our platform collects should allow the construction and fitting of predictive models of germination dynamics in polymicrobial communities.

There are multiple directions in which this work can be extended. The compounds mediating positive and negative interactions can be chemically characterized. The automated methodology for image capture and analysis can be harnessed to further explore diversity by studying larger networks and to screen for mutants with different production or susceptibility profiles in order to investigate the genetic underpinnings of germination interactions and stochastic germination behavior. On the ecological and evolutionary side, it would be important to perform microcosm experiments demonstrating or rejecting the adaptive nature of germination interaction and stochastic germination. These can be combined with mathematical modeling to understand what types of interactions and what types of stochastic behavior are favored in different circumstances and whether diverse strategies can coexist in a single environment.

Improved understanding of how germination is modulated can lead to practical applications. For example, compounds stimulating exit from dormancy can be combined with antibiotics to treat infections that are persistent due to bacterial dormancy. Such compounds can also be useful in resuscitating and culturing dormant bacteria from natural environments.

## Methods

### Preparation of *Streptomyces* Spores

*S. coelicolor* M145 and *S. spp*. GrRd7 were streaked and grown on ISP2 agar medium (4 g Glucose, 4 g Yeast extract, 10 g Malt extract, 15 g Agar in 1 L ddH_2_O), *S. viridochromogenes* B1511 was streaked and grown on 5006 agar medium (3 g Sucrose, 15 g Dextrin, 1 g Meat extract, 2 g Yeast extract, 5 g Tryptone soy broth, 0.5 g NaCl, 0.5 g K_2_HPO_4_, 0.5 g MgSO_4_ × 7H_2_O, 0.01 g FeSO_4_ × 7H_2_O, 20 g Agar in 1 L ddH_2_O), and *S. venezualae* ISP5230 was streaked and grown on MYM (4 g Maltose, 4 g Yeast extract, 10 g Malt extract, 18 g Agar in 1 L ddH_2_O) agar medium at 28 °C for one week. Single colonies were picked for each strain and streaked across the respective agar medium to generate a sporulation lawn. After one week growing at 28 °C, spores were harvested and suspended in cool Tris-Tween buffer (0.05 M Tris, 0.001% Tween80, pH 7.3). To remove germinated spores or mycelia, the crude spore suspension was filtrated through a 5 µm nylon membrane, then washed twice by cool Tris-Tween buffer and stored at 4 °C for immediate usage or in saline-glycerol buffer (0.85% saline and 25% glycerol) in −80 °C for long term storage.

### Construction of Fluorescent Spores

*sigE* is an ECF (Extracytoplasmic Function) RNA polymerase sigma factor involved in cell envelope stress responses of *S. coelicolor* ^33^. Its promoter was amplified from chromosomal DNA of *S. coelicolor* M145 using forward and reverse oligonucleotides: 5′ CGGCTG***GATATC***CCGCTGAGCTGACGCACAGTCG 3′ and 5′ CGAGCA***TCTAGA***CCTCGCCCATGTCAACCGCCTTCC 3′ (EcoRV and XbaI sites italicized and bolded) to build fluorescent reporters expressed in *S. coelicolor* M145, *S. sp*. GrRd7 and *S. viridochromogenes* B1511. For *S. venezualae* ISP5230, promoter of *SCO5466*, a putative cell wall hydrolase of *Streptomyces* ^34^ was amplified using 5′ TCCCGAC***GATATC***CGTCGAGTCGTCCTTCGAACC 3′ and 5′ TGGCGGA***GG***-***ATCC***CTGGTACGCGCTGACGTCGAT3′ (EcoRV and BamHI site italicized and bolded). To build the green fluorescent reporter, the amplified promoter products were digested with respective restriction enzymes (EcoRV and XbaI for *sigE* promoter, EcoRV and BamHI for *SCO5466* promoter) and were ligated into the integrating plasmid pIJ8660 that harbors a promoterless green fluorescent gene *egfp*. The resulting plasmids were p*sigE*p-*egfp* and p*SCO5466*p-*egfp*. To build the red fluorescent reporter, the red fluorescent gene *dTomato* was amplified from the plasmid p67T1 with forward and reverse oligonucleotides 5′ GGGACTTC***CATATG***GTGAGCAAG-GGCGAGGAGGTCATC 3′ and 5′ CAACCAAGCA***GCGGCCGC***CTACTTGTACAGCTCGTCCATGCC 3′ (NdeI and NotI site italicized and bolded), and then was used to replace the green fluorescent gene *egfp* located between the NdeI and NotI sites in both p*sigE*p-*egfp* and p*SCO5466*p-*egfp*. The resulting plasmids were p*sigE*p-*dt* and p*SCO5466*p-*dt*.

The p*sigE*p-*egfp* and p*sigE*p-*dt* were introduced into *S. coelicolor* M145*, S. viridochromogenes* B1511 and *S. spp*. GrRd7, and the p*SCO5466*p-*egfp* and p*SCO5466*p-*dt* were introduced into *S. venezualae* ISP5230 by conjugation through the methylation deficient *E. coli* strain ET12567-PUZ8002 (Supplementary Table 2). The bacteria mix was grown on MS agar for 16 hours and overlaid by apramycin (50 µg/ml) and nalidixic acid (25 µg/ml). Candidate exconjugants were further selected by growing on agar media as described above in the Preparation of spores section with added apramycin (50 µg/ml). Single colonies were picked up and grown to a lawn in the presence of apramycin (50 µg/ml) at 28 °C for spore harvest. The purified candidates of fluorescent spores were checked under microscope for quality and spore density and stored as described previously.

### Setting Up and Microscopic Recording of Germination

A 22 mm × 76 mm × 2 mm silicone (polysiloxane) sheet (McMaster-Carr) with 6 punching holes (6 mm diameter) was glued onto a glass slide of the same size to make the microscopic device for germination. Each well of the device was filled with 80 µl nutrient broth agar (2% NB is made of 0.16 g Nutrient Broth powder and 1.2 g purified agar in 100 ml ddH_2_O, 10% NB is made of 0.8 g nutrient broth powder and 1.2 g purified agar in 100 ml ddH_2_O). Non-interaction experiments used 2% NB. Spores from the storage stock (~10^9^–10^10^ spores/ml by colony count on petri-dish) were washed once and serially diluted. 5 µl of each diluted sample was loaded on the microscopic germination device to check the spore density. An estimated spore density, spores per field of view (spores/f.v.) was calculated automatically by a script and averaged from 10 fields of view for each dilution. Then the spore sample was properly diluted in ddH_2_O (or germination supernatant or other buffer) to get the density needed (e.g. ~100 spores/f.v.) and inoculated into each well and air-dried in a laminar flow hood for 1 hour. The device was covered by three 22 mm × 22 mm coverslips and loaded on the stage of Nikon Ti-E inverted fluorescent microscope. The germination was recorded at 28 °C for 10–24 hours. Images were taken for phase contrast and fluorescent channels every hour (20x objective, 70 ms exposure for phase contrast and 2 s exposure for fluorescence). For each well, 10 to 40 non-overlapping fields of view were filmed. Each field of view was 1350 × 1000 pixels with each pixel corresponding to 0.15 µm. For each field of view, movie frames from different time points were aligned using NIS-Elements Nikon software to compensate for small shift during recording.

### Germination Interaction by Germination Supernatant

Fresh non-fluorescent spores of each *Streptomyces* species were harvested from sporulation lawns growing on solid agar media as described above and inoculated into 2 ml 2% NB liquid medium. The density was such that 5 µl pipetted onto an agar well resulted in around 200 spores per field of view (spore/f.v.). The culture was growing in a 28 °C shaker at 200 rpm and the germination status was checked every hour. At 4 h for *S. venezuaelae* ISP5230 and *S. viridochromogenes* B1511, 2 h for *Streptomyces spp*. GrRd7, and 16 h for *S. coelicolor* M145 after inoculation, the swelling or tiny sprouts were observed for most spores. The germination culture was centrifuged at 10,000 rcf for 10 min at 4 °C to remove spores and germlings, and the supernatant was transferred and stored at 4 °C for intermediate use or at −20 °C for future use. The testing fluorescent spores were diluted to a density of around 100 spores/f.v. and resuspended in the supernatant of the germination culture (germsup) or the reference buffer (ddH_2_O or 2% NB liquid media). 5 µl of spores treated by germsup, ddH_2_O reference or 2% NB reference were inoculated in duplicate onto wells containing 10% NB agar, and germination was recorded as described above.

### Germination Interaction by Co-culturing Two Different *Streptomyces* Species

*Streptomyces* spores of two species expressing red or green fluorescence were mixed in 1:1 ratio in a combined density of around 100 spores/f.v. As a reference, each species was grown by itself at the same density as in the mix culture (around 50 spores/f.v.). 10% NB agar was used as the media inside each well. Each condition was present in duplicate.

### Image Processing and Quantification of Germination

The time-lapse movies were processed by custom MATLAB scripts, which are available upon request. A Gaussian filter was applied to the fluorescent channel(s) and the median and standard deviation of the distribution of pixel intensities for each image was calculated. Points which were not significantly different than the background (typically intensity that was less than three standard deviations from the median) were set to zero intensity and a watershed algorithm was used to segment touching spores by setting the intensity along watershed boundaries to zero. The so processed fluorescent channel was then converted to a binary image by setting all non-zero intensities to one and the area, perimeter, and centroids of all connected regions were quantified. Any connected regions significantly different than spores by size were then removed. The spore coordinates were calculated as the centroids of the connected regions at the first timepoint. The distances between spores within a time frame and between time frames were used to compensate for occasional small movements of spores along the surface.

Once the position of a spore over time was traced, the fluorescent and phase contrast channels were combined to extract the boundaries of the spore and, if the spore germinated, the mycelium network. Germination was then determined by the onset of increase in the major axis of this contour. While the automatic processing was highly reliable, we still manually verified all the data presented in this paper to make sure that none of the effects we reported were due to rare imaging or image-processing artifacts. This was done spore by spore by displaying all time points of the surrounding of an individual spore on the screen along with the automatically derived annotation and allowing the user to approve or occasionally correct the annotation. The custom software we employed allowed manual verification at a rate of one spore per 2 seconds and the order of spores scored for each experiment was randomized to avoid biases.

### Quantification and Statistics of Germination Curves

The value of the germination curve at a timepoint is defined as the number of spores that germinated at that timepoint or earlier divided by the total number of tracked spores. The germination curves were built by combining data from all fields of views across one or two different wells.

To visually compare differences between germination curves across different conditions (e.g. nutrient level, spore density and spore age), for each curve we computed lower and upper boundaries corresponded to a 95% confidence interval. This was done by interpreting the germination curve as a cumulative distribution function for the stochastic germination times. This cumulative distribution was then used to generate 1000 random sets of germination times and, from there, 1000 randomized germination curves with the same sample size. For each timepoint we then found the lower and upper bounds containing 95% of the randomized curves.

To determine whether a difference between two germination curves is statistically significant, a two-sample Kolmogorov-Smirnov test (K-S test) was applied to the cumulative probability distributions indicated by the curves. The p-values from these comparisons are presented.

For the comparison between doublets (two touching spores) and singles (a spore which is at least 30 pixels separated from other spores) in Fig. 3c, the germination curve for the singles was used as a cumulative distribution function for a null model. This null model was used to generate sets of random germination times whose size matched the number of spores in the doublets. These random sets were then used to construct a cloud of randomized curves (null expectation for doublets based on the germination of singles) representing the cumulative fraction of pairs of spores for which both spores within a pair have germinated by a given time. For each randomized curve (thin black lines on Fig. 3c), we computed the maximal vertical distance to the mean curve expected from the null model (thick black line). The p-value was calculated as the fraction of randomized curves for which the maximum vertical distance was larger than that for the actual doublet data (red line).

### Quantification and Statistics of Growth Rates and Mycelium Stalling Fractions

A growth curve of an individual spore was quantified as the area (number of pixels) occupied by the spore or the mycelium that emerged from this spore. The germling contour was recognized by an automatic script and followed in time until it touched another spore or mycelium. To remove occasional image processing errors, the contours were manually verified by displaying movie frames of individual spores and their surroundings on the screen along with the automatically drawn contours and allowing the user to approve or pick the last frame with the correct contour identification (Fig. S1). Since mycelium thickness is roughly constant, the mycelium area is proportional to the total length of the filaments in the mycelium network.

If the rate of change of the area after germination fell below a threshold (10 pixels per hour) for more than two hours, the mycelium was annotated as having stopped growing, i.e. “stalled”. The number of stalled mycelium was divided by the total number of germinated spores to obtain the fraction of stalling mycelium. Error bars (Figs 2c and 5III) indicate 95% confidence intervals based on a binomial distribution with same mean and sample size. Fisher’s exact test was used to evaluate the statistical significance of differences of the stalling fraction between conditions.

The initial growth rate was calculated for non-stalling spores that continuously grew for at least 5 hours (5 time points) after the germination and whose final mycelium area was at least twice of the original spore area. The natural logarithm of these areas was fitted by a linear regression, and the initial growth rate, *g*, was set to the slope of the fitted line. The two-sample Kolmogorov-Smirnov test was used to compare growth rate distributions.

### Additional Information about Figure Data

All data for a given condition were collected from one or two wells with 10 or more fields of views per well. All qualitative conclusions were confirmed by at least one more independent experiment with different spore batches that were harvested on different days. Below we provide sample size data and p-values that were not included in the figure captions.

Figure 2: Each strain was randomly scored from 15 fields of view in one well. Sample sizes (b), n_GrRd7_ = 481, n_B1511_ = 553, n_ISP5230_ = 411, n_M145_ = 520; Sample sizes (c,d), _GrRd7_ = 312, n_B1511_ = 102, n_ISP5230_ = 194, n_M145_ = 125.

Figure 3: Each strain was randomly scored from 10 fields of view in one well. Sample sizes, n_blue_ = 326, n_red_ = 263, n_yellow_ = 148, n_purple_ = 130. *p* values for comparison between curves (a) blue vs. red < 10^−4^, blue vs. yellow < 10^−16^, red vs. yellow < 10^−5^; (c) from left to right panel, *p* = 0.002, 0.16, 0.94.

Figure 4(b) All buffer and germsup treated strains were randomly scored from 20 fields of view in two wells and repeated in at least one more independent experiment. Only interactions significant in all independent experiments were highlighted as interactions. Sample sizes for column 1 (GrRd7 germsup), n_GrRd7_buffer_ = 235, n_GrRd7_germsup_ = 187, n_B1511_buffer_ = 485, n_B1511_germsup_ = 457, n_ISP5230_buffer_ = 230, n_ISP5230_germsup_ = 500, n_B1511_buffer_ = 315, n_B1511_germsup_ = 330; for column 2 (B1511 germsup), n_GrRd7_buffer_ = 312, n_GrRd7_germsup_ = 405, n_B1511_buffer_ = 339, n_B1511_germsup_ = 336, n_ISP5230_buffer_ = 215, n_ISP5230_germsup_ = 288, n_B1511_buffer_ = 407, n_B1511_germsup_ = 471; for column 3 (ISP5230 germsup), n_GrRd7_buffer_ = 324, n_GrRd7_germsup_ = 318, n_B1511_buffer_ = 336, n_B1511_germsup_ = 321, n_ISP5230_buffer_ = 270, n_ISP5230_germsup_ = 264, n_B1511_buffer_ = 208, n_B1511_germsup_ = 221; for column 4 (M145 germsup), n_GrRd7_buffer_ = 227, n_GrRd7_germsup_ = 347, n_B1511_buffer_ = 371, n_B1511_germsup_ = 355, n_ISP5230_buffer_ = 237, n_ISP5230_germsup_ = 240, n_B1511_buffer_ = 330, n_B1511_germsup_ = 354. *p* values for highlighted significant interactions: for self-promotion of ISP5230 (row 3, column 3), *p* = 10^−4^; for promotion of M145 by GrRd7 germsup (row 4, column 1), *p* < 10^−12^; for inhibition of M145 by B1511 germsup (row 4, column 2), *p* < 10^−60^; for promotion of M145 by ISP5230, *p* < 10^−28^; for self-inhibition of M145 (row 4, column 4), *p* < 10^−5^. We also observed shift of curves in one independent experiment shown in Fig. 4 for the following interactions: Earlier germination of GrRd7 by M145 germsup (row 1, column 4), *p* < 10^−27^; Earlier germination of B1511 by M145 germsup (row 2, column 4), *p* < 10^−27^; Delayed germination of B1511 by its own germsup (row 2, column 2), *p* < 10^−13^. However, we did not claim these three interactions as they were not reproducible in other experimental repeats. (c) Top, spores stored for different days were randomly scored from 10–20 fields of view of one well and repeated in three independent experiments using different spore batches. Sample sizes: n_day 1_ = 411, n_day 2_ = 263, n_day 3_ = 487, n_day 5_ = 418, n_day 7_ = 197. *p* values between curves: day 1 vs. day 2 < 10^−23^, day 1 vs day 3 < 10^−45^, day 1 vs day 5 < 10^−34^, day 2 vs day 3 = 0.13, day 2 vs day 5 = 0.994, day 3 vs day 5 = 0.34. Bottom, old spores treated by buffer and germsup form fresh spores were randomly scored from 20 fields of view of two wells and repeated once in a different experiment. Sample sizes: n_buffer_ = 843, n_germsup_ = 680. *p* < 10^−25^.

Figure 5: These are the same data as in Fig. 4 but analyzed for mycelium growth. Mycelium sample sizes (a) n_buffer_ = 17, n_germsup_ = 87; (b) n_buffer_ = 81, n_germsup_ = 122; (c) n_buffer_ = 258, n_germsup_ = 87. Influence of germsup on initial growth rate of treated spores (column II): ISP5230 germsup promote growth rate of its old spores, *p* = 0.0091; ISP5230 germsup did not affect growth rate of M145, *p* = 0.0681; B1511 germsup promote growth rate of M145, *p* = 3 × 10^−6^. Influence of germsup on stalling fraction of treated spores (column III): ISP5230 germsup on its old spores, *NA*; ISP5230 germsup had no effect on stalling fraction of M145, *p* = 0.8695; B1511 germsup reduced stalling fraction of M145, *p* = 0.037.

Figure 6(b) Spores in mono or mix culture were randomly scored from 40 fields of views of two wells and repeated once in an independent experiment. Only interactions significant in all independent experiments were highlighted and claimed in the result. Sample sizes: ISP5230 vs. M145, n_mix_red_ = 303, n_mix_green_ = 638, n_mono_red_ = 336, n_mono_green_ = 503. ISP5230 vs. GrRd7, n_mix_red_ = 285, n_mix_green_ = 388, n_mono_red_ = 332, n_mono_green_ = 316. ISP5230 vs. B1511, n_mix_red_ = 258, n_mix_green_ = 324, n_mono_red_ = 295, n_mono_green_ = 261. B1511 vs. GrRd7, n_mix_red_ = 488, n_mix_green_ = 478, n_mono_red_ = 466, n_mono_green = _395. B1511 vs. M145, n_mix_red_ = 504, n_mix_green_ = 520, n_mono_red_ = 508, n_mono_green_ = 529. M145 vs. GrRd7, n_mix_red_ = 496, n_mix_green_ = 487, n_mono_red_ = 509, n_mono_green_ = 301. *p* values for highlighted significant interactions: ISP5230 was inhibited by co-cultured M145, *p* < 10^−26^; M145 was inhibited by co-cultured B1511, *p* < 10^−15^.

## Electronic supplementary material

Supplementary Information
