# Supplementary material for: Phenotypic variability and community interactions of germinating Streptomyces spores
Source: Sci Rep. 2017 Apr 6;7:699. doi: 10.1038/s41598-017-00792-7 (PMC5429633; doi:10.1038/s41598-017-00792-7)
Supplement: Supplementary file 1 — Supplementary Information [file 41598_2017_792_MOESM1_ESM.pdf]

Supplementary Information for

Phenotypic variability and community interactions of germinating *Streptomyces* spores

Ye Xu[1] & Kalin Vetsigian[1]

[1]Department of Bacteriology and Wisconsin Institute for Discovery, University of Wisconsin-Madison, Wisconsin, 53715, USA.

Corresponding Author:

Prof. Kalin Vetsigian

kalin@discovery.wisc.edu

Tel: 608-316-4670

Supplementary Figures:

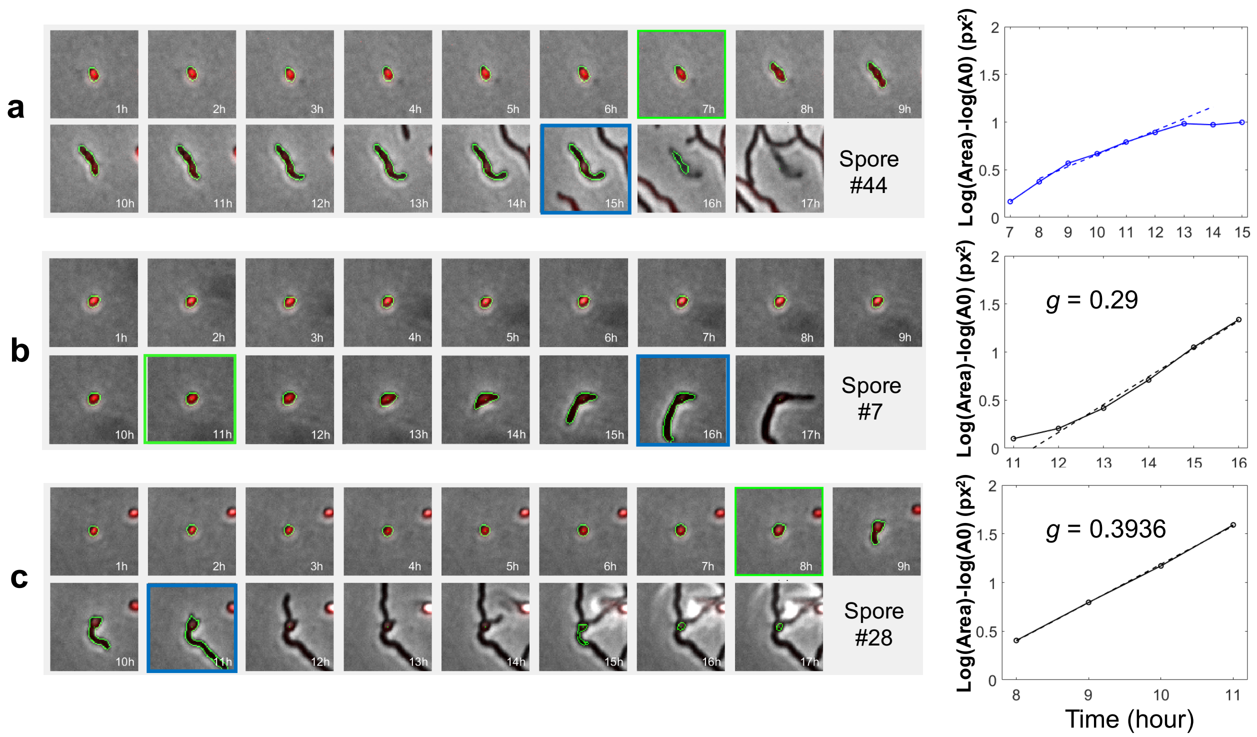

Fig. S1. Examples of mycelium growth from the field of view in Fig.1a. Spores were examined in a square neighborhood of the shown size. Green contours outline the area occupied by a spore or its mycelium network. Blue frames indicate the last time point for which the contour fits inside the square neighborhood or for which the contour was accurately identified (manually verified). Green frames show the scored germination time. The growth rate was quantified as the slope of the line fitted to the logarithm of the area versus time. (**a**) The growth of spore #44 stalled at around 12 hour, as indicated by the plateauing of the area, and died. (**b**) Spore #7 did not stall and grew at a moderate speed. (**c**) Spore #28 did not stall and grew fast.

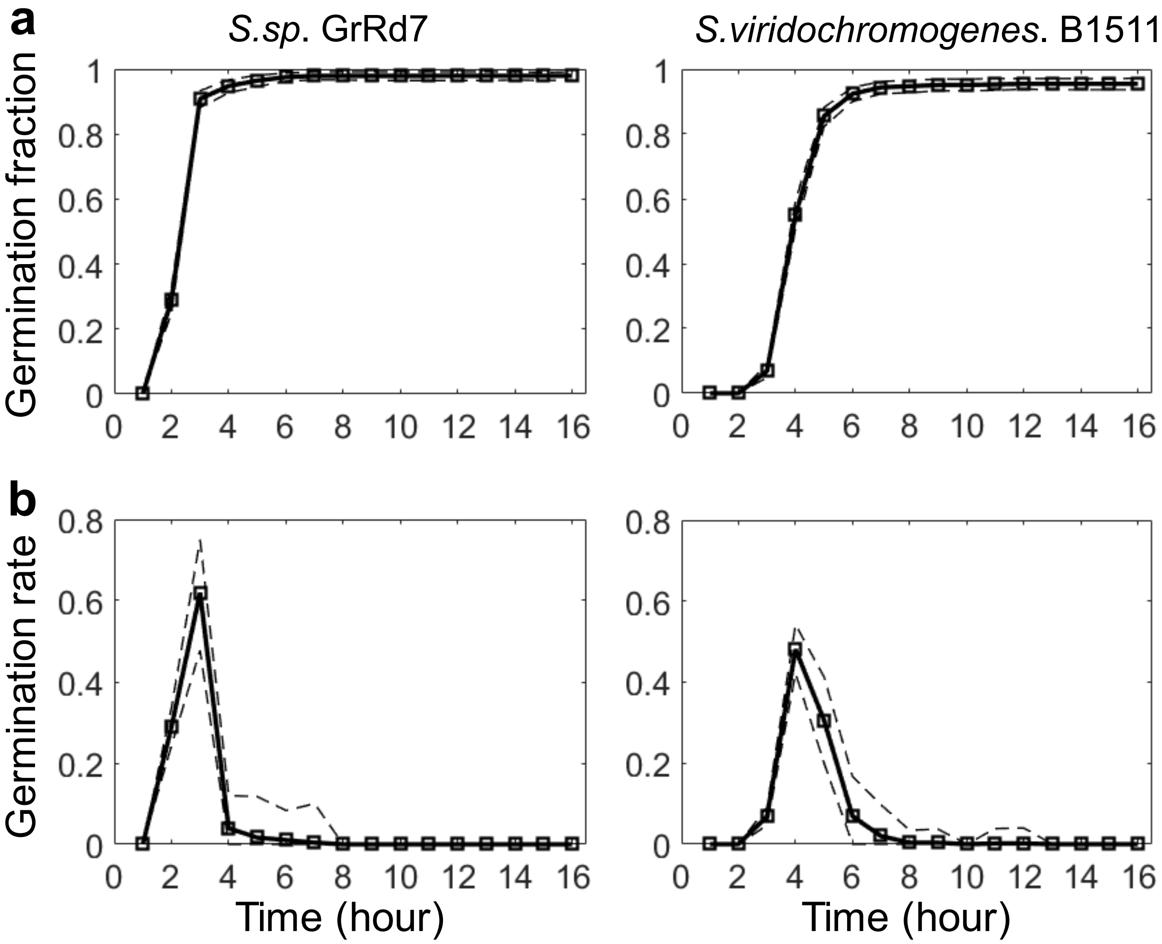

Fig. S2. Robust germination of *S.sp.* GrRd7 and *S.viridochromogenes* B1511. (**a**) The germination curves of the two strains exhibited an exponentially decreasing fraction of non-germinated spores, and the final germination fraction of both was close to 1. (**b**) The germination rate was quantified as the fraction of germinated spores per hour. The rate of germination peaks shortly after the onset of germination and then monotonically decreases. The 95% confidence intervals are indicated as dashed lines. Sample sizes: $n_{GrRd7} = 481$, $n_{B1511}=553$.

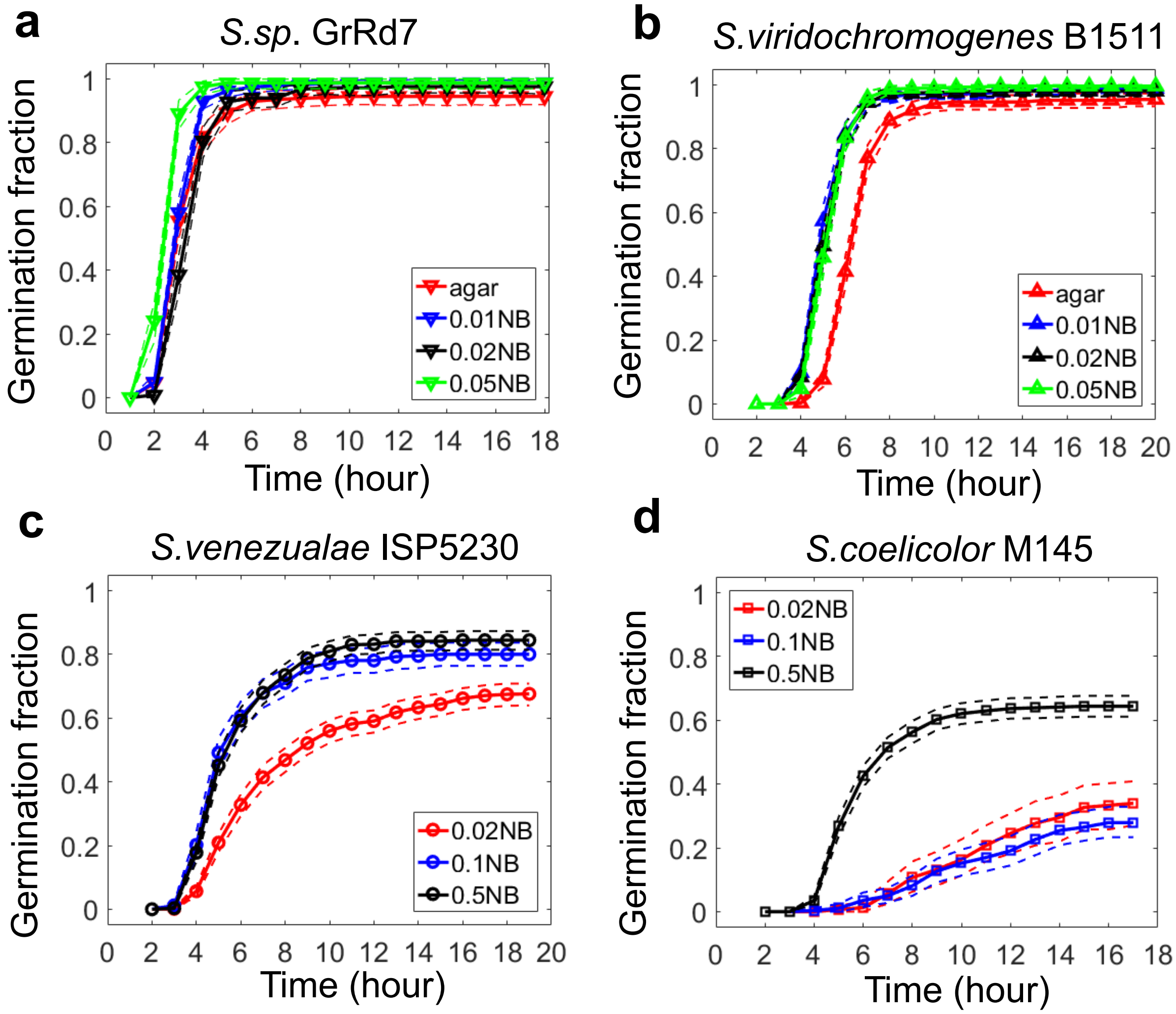

Fig. S3. Germination curves at different nutrient concentrations. 0.01NB stands for hundred times diluted nutrient broth, etc. The dashed lines indicate 95% confidence intervals. Sample sizes, low to high nutrient concentration: n_{GrRd7} = 312, 353, 252, 266. n_{B1511} = 376, 366, 309, 296. $n_{ISP5230}$ = 775, 411, 631. n_{M145} = 159, 283, 326. **(a, b)** *S.sp.GrRd7* and *S.viridochromogenes* B1511 germinate robustly even on purified agar (red). The lag time decreases with the increase of the nutrient concentration in the agar for *S.viridochromogenes* B1511 (red and blue curves differ significantly, $p < 10^{-40}$). **(c, d)** Only a fraction of the spores of strains *S.venezualae* ISP5230 and *S.coelicolor* M145 germinate. The fraction of germinating spores increases with nutrient concentration. For (c), $p<10^{-18}$ for the difference between red and blue curves. For (d), $p< 10^{-25}$ for the difference between red and black curves.

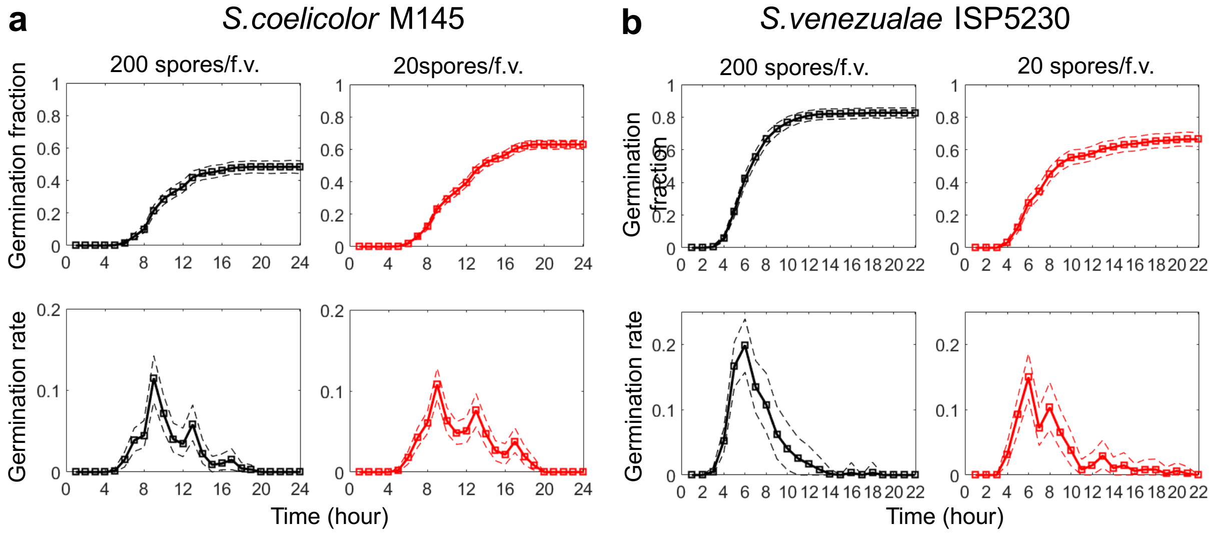

Fig. S4. Non-trivial temporal structure of germination curves. Top panels show germination curves and bottom panels show calculated germination rates (fraction of germinated spores per hour). The 95% confidence intervals are indicated as dashed lines. **(a)** *S.coelicolor* M145 exhibits a fluctuating germination rate both at high (left panels) and low (right panels) densities. Sample sizes: n_{high} = 670, n_{low} = 1248. **(b)** *S.venezualae* ISP5230 exhibits a shift to higher and single peaked germination rate at high spore densities, which agrees with its self-promotion of germination. Sample sizes: n_{high} = 594, n_{low} = 474.

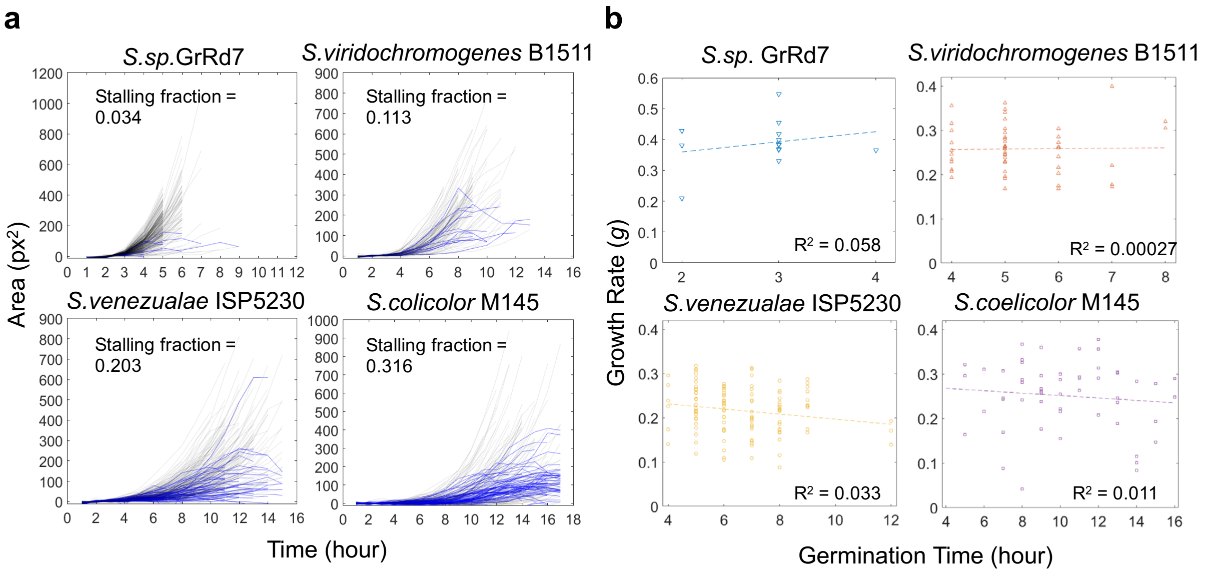

Fig. S5. Growth bistability after germination and relationship between growth rate and germination time of 4 Streptomyces strains. (a) The fate of a spore after germination is either an exponential mycelium growth (black curve) or mycelium that stops growing (stalls) while the network is still small (blue curve). The area of spore and mycelium is plotted over time. Sample sizes: n_{GrRd7} = 113, n_{B1511} = 138, $n_{ISP5230}$ = 219, n_{M145} = 205. **(b)** The initial growth rate of all 4 Streptomyces was not correlated with its germination time. The Pearson correlation coefficient R^2 is shown.

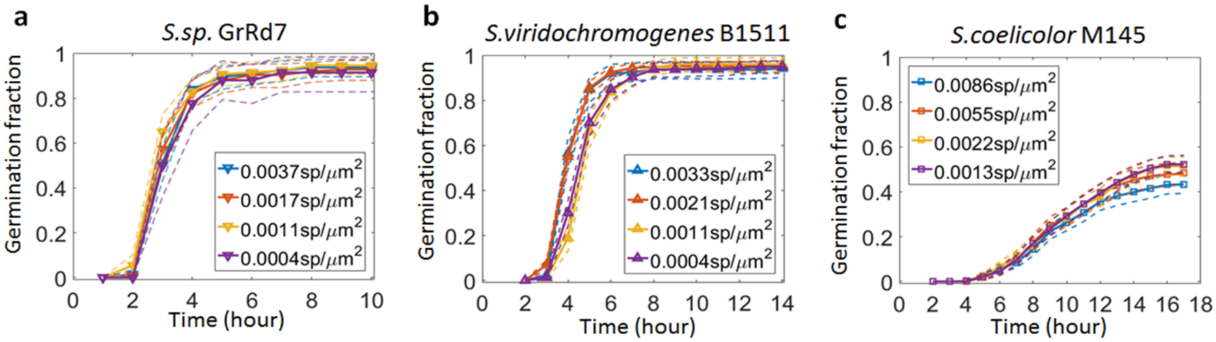

Fig. S6. Germination curves at different spore densities for 3 of the strains. **(a, b)** *S.sp.GrRd7* and *S.viridochromogenes* B1511 germinate robustly at any density. For (a), the differences between curves are not statistically significant. For (b)**,** yellow and purple curves shifted to the right, *p* values between curves: blue vs. red = 0.9444, blue vs. yellow $<10^{-10}$, blue vs. purple $<10^{-5}$, red vs yellow $<10^{-5}$, red vs purple, 10^{-8}, yellow vs purple = 0.1479. **(c)** *S.coelicolor* M145 shows slightly higher germination fraction at the lowest spore density compared to highest density. *p* value between blue and purple = 0.0173. The 95% confidence intervals were indicated as dashed lines. Sample sizes, from high to low: n_{GrRd7} = 143, 124, 128, 58. n_{B1511} = 172, 253, 176, 237. n_{M145} = 520, 424, 611, 641.

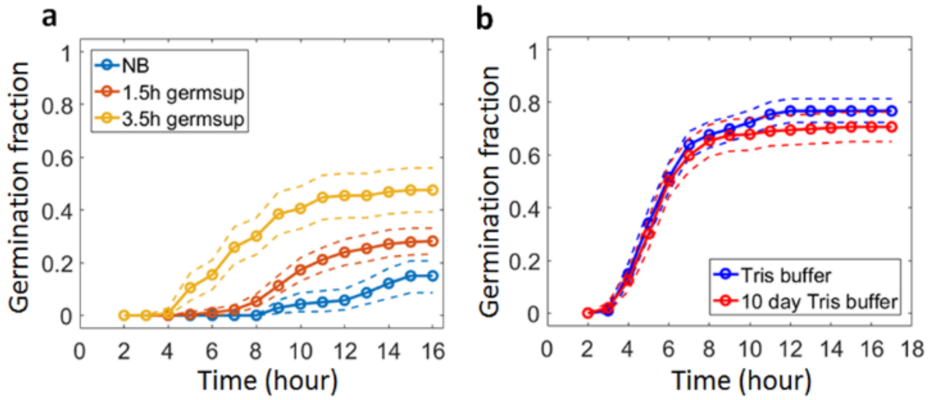

Fig. S7. Effect on germinaton of *S.venezualae* ISP5230 by germination supernatants obtained from different time points and a prolonged storage buffer. The 95% confidence intervals are indicated as dashed lines. **(a)** Germination supernatant (germsup) was obtained by incubating spores in 0.02NB for different lengths of time and applied to 7-day old spores. Spores incubated for 1.5 hours did not show any sprouts but the germsup from this time point mildy increased germinability of old spores (p = 0.0033). Germsup from spores incubated for 3.5hour, at which point germ tubes started to form, boosted germination to even higher level ($p<10^{-9}$). Sample sizes: n_{NB} = 140, $n_{1.5h}$ = 284, $n_{3.5h}$ = 143. (**b**) Fresh spores were treated by fresh storage buffer and buffer from prolonged storage of spore stock and showed no significant difference between the two (p = 0.4412). Thus, there was no detectable chemical leakage of germination promotion compounds to the storage buffer, which could have explained the decreased germibility of old spores. Sample sizes: n_{Tris} = 322, $n_{10day\text{-}Tris}$ = 249.

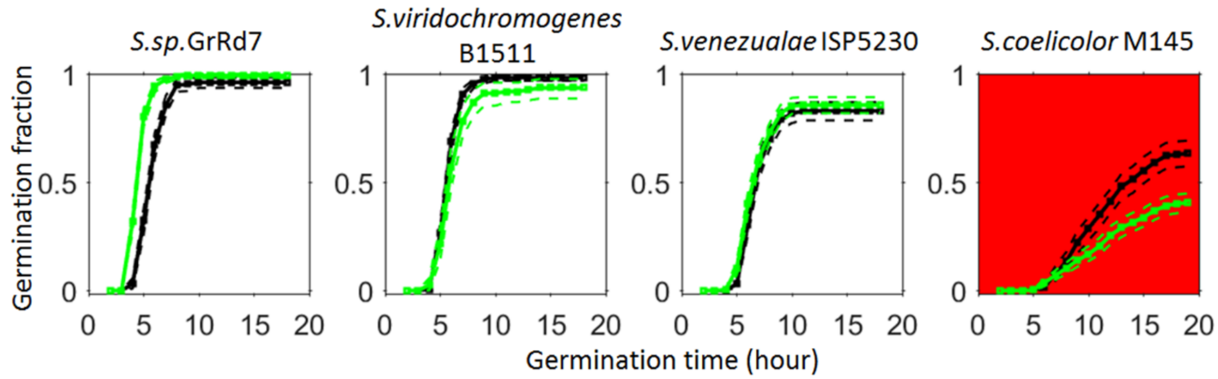

Fig. S8. Effect of germination supernatant from *S.coelicolor* M145 obtained from spores germinating in higher resource concentration. Germination supernatant was obtained by incubating M145 spores in 0.1NB for 7 hours instead of 0.02NB for 16 hours. This germsup exhibited strong self-inhibitory activity ($p < 10^{-7}$) even despite the fact that the difference in added resources between germsup (green) and H_2O (black) was much more substantial. The stronger effect can be attributed to the more robust germination of M145 in 0.1NB liquid. The 95% confidence intervals are indicated as dashed lines. Sample sizes, reference(black) and germsup (green): n_{GrRd7} = 237, 337. n_{B1511} = 338, 222. $n_{ISP5230}$ = 321, 324. n_{M145} = 269, 474.

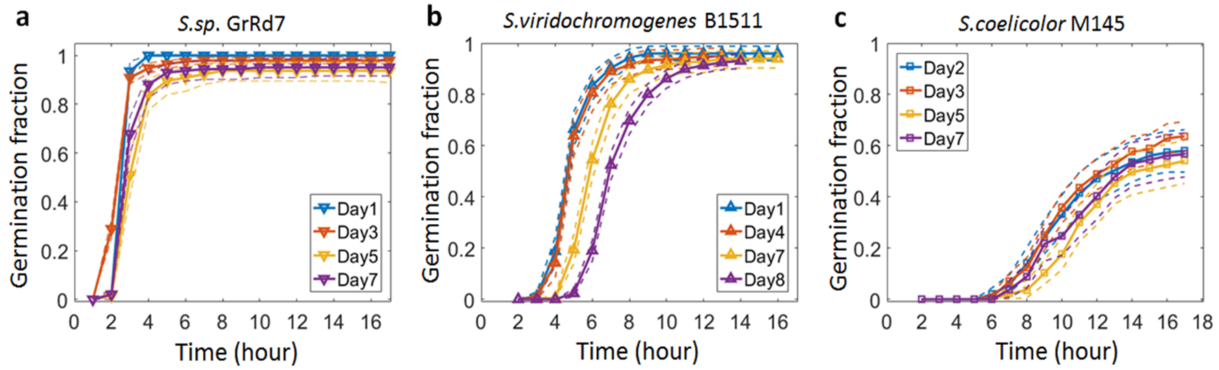

Fig. S9. Germination curves of spores stored for different periods for 3 of the strains. **(a)** Longer storage had little effect on the germination of *S.sp.GrRd7*. The older spores (Day5 and Day 7) had less final germination fraction (p between Day3 and Day5$<10^{-5}$). **(b)** The germination onset of *S.viridochromogenes* B1511 was delayed when it was stored for longer period of time (curve shifted to the right, $p<10^{-13}$ between Day4 and Day7, and $p<10^{-12}$ betweenDay7 and Day8), but the robust germination mode and the final fraction of germinated spores were not affected. **(c)** Germination curves of *S.coelicolor* M145 from different days were not significantly different from each other, though the curve for Day5 is slightly different than that for Day3 (p=0.0044). Dashed lines indicate 95% confidence intervals. Sample sizes, from young to old: n_{GrRd7} = 124, 181, 143,143. n_{B1511} = 176, 155, 165, 347. n_{M145} = 157, 245, 137, 134.

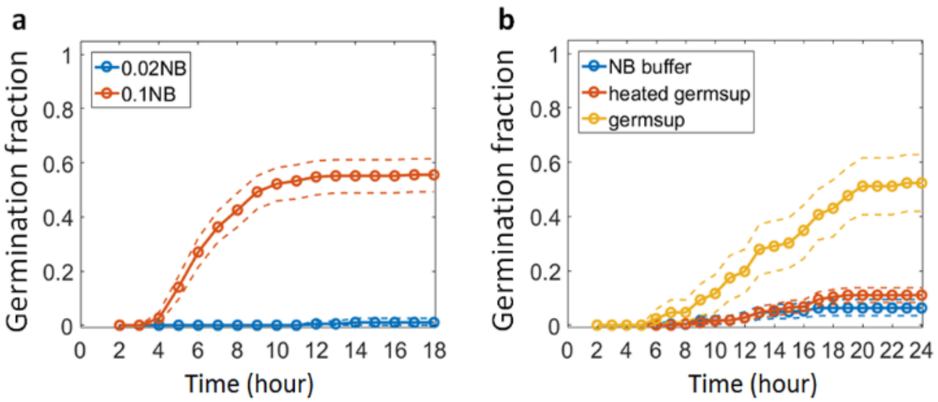

Fig. S10. Additional experiments with aged spores of *S.venezualae* ISP5230. The 95% confidence intervals were indicated as dashed lines. **(a)** The germinability of old spores can be recovered upon increasing the nutrient concentration ($p < 10^{-30}$). Sample sizes: $n_{0.02NB} = 197$, $n_{0.1NB} = 270$. **(b)** The germsup from *S.venezualae* ISP5230 lost its self-promotional activity (yellow and blue curve differe significantly, $p < 10^{-12}$), upon heating for 10 min at 100°C (red and blue curves are not significantly different, p=0.82). Sample sizes: $n_{buffer} = 286$, $n_{germsup} = 244$, $n_{heated\ germsup} = 106$.

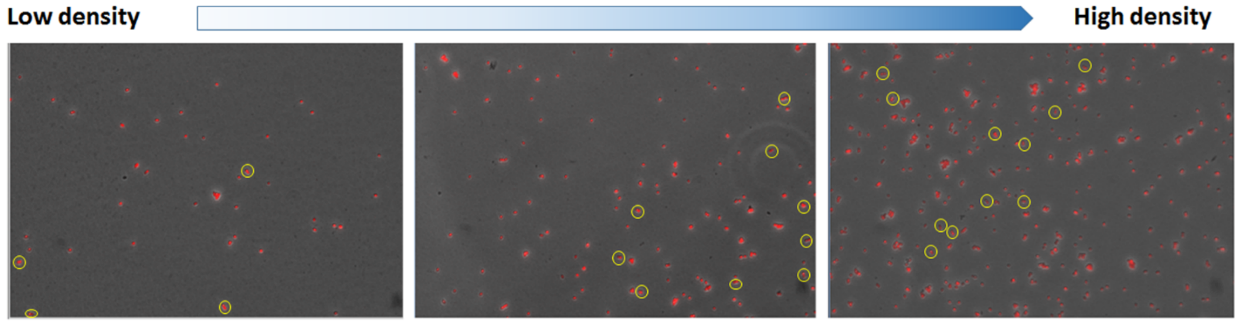

Fig. S11. Examples of clusters of two or more spores in environments of low to high spore densities. The doublets are highlighted by yellow circles.

Supplementary Table:

Table S1. Bacterial strains, plasmids and constructs

Bacterial strains			
**Strain #**	**Strain name**	**Description**	**Source**
**1**	*Streptomyces viridochromogenes* NRRL B1511		ARS (NRRL)
**2**	*Streptomyces sp.* GrRd7	Wild soil isolate from Devil's Lake Park, WI	This work
**3**	*Streptomyces venezualae* ISP5230		ARS (NRRL)
**4**	*Streptomyces coelicolor* M145	*S.coelicolor*, $SCP1^{-}$, $SCP2^{-}$	J. Nodwell lab,

			McMaster University
**5**	*Escherichia.coli DH5α*	Cloning host	(Taylor et al. 1993)
**6**	*Escherichia.coli* ET12567/pUZ8002	Methylation deficient and conjugal donor strain carrying the non-transmissible pUZ8002 that provides the *in trans* transfer function	(Flett et al. 1997)
	**Plasmids and constructs**		
**Plasmid #**	**Plasmid or construct name**	**Description**	**Source or reference**
**1**	pIJ8660	*Streptomyces* integrative vector containing promoterless *eGFP* gene adapted to the codon usage of *Streptomyces*; Apramycin^r.	(Sun et al. 1999)
**2**	p67T1	pMMB66EH-based construct carrying 718-bp BamHI-HindIII *dTomato* DNA; Apramycin^r; constitutive pigment production	(Singer et al. 2010)
**4**	p*sigEp-egfp*	pIJ8660 containing 344bp promoter region of *sigE*, transcriptionally fused with *eGFP*	This work
**5**	p*sigEp-dt*	p*sigEp-egfp* in which the *eGFP* between NdeI and NotI restriction sites was replaced by a 718bp fragment of *dTomato*	This work
**6**	p*SCO5466p-egfp*	pIJ8660 containing 365bp promoter region of SCO5466, transcriptionally fused with *eGFP*	This work

References

Flett, F., Mersinias, V. & Smith, C.P., 1997. High efficiency intergeneric conjugal transfer of plasmid DNA from Escherichia coli to methyl DNA-restricting streptomycetes. *FEMS Microbiology Letters*.

Singer, J.T. et al., 2010. Broad-Host-Range Plasmids for Red Fluorescent Protein Labeling of Gram-Negative Bacteria for Use in the Zebrafish Model System. *Applied and Environmental Microbiology*, 76(11), pp.3467–3474.

Sun, J. et al., 1999. Green fluorescent protein as a reporter for spatial and temporal gene expression in Streptomyces coelicolor A3 (2). *Microbiology*, 145(9), p.2221.

Taylor, R.G., Walker, D.C. & McInnes, R.R., 1993. E. coli host strains significantly affect the quality of small scale plasmid DNA preparations used for sequencing. *Nucleic Acids Research*, 21(7), pp.1677–1678.
